# Machine Learning for Understanding and Predicting Injuries in Football

**DOI:** 10.1186/s40798-022-00465-4

**Published:** 2022-06-07

**Authors:** Aritra Majumdar, Rashid Bakirov, Dan Hodges, Suzanne Scott, Tim Rees

**Affiliations:** 1 Department of Rehabilitation and Sport Science, Faculty of Health and Social Sciences, Bournemouth University, Dorset House, Talbot Campus, Fern Barrow, Poole, BH12 5BB UK; 2 Department of Computing and Informatics, Faculty of Science and Technology, Bournemouth University, Dorset House, Talbot Campus, Fern Barrow, Poole, BH12 5BB UK; 3 AFC Bournemouth, Vitality Stadium, Dean Court, King’s Park, Bournemouth, BH7 7AF, UK; 4 Newcastle United Football Club, St. James’ Park, Strawberry Place, Newcastle upon Tyne, NE1 4ST UK

**Keywords:** Training load, Football injuries, Machine learning, Injury prediction

## Abstract

Attempts to better understand the relationship between training and competition load and injury in football are essential for helping to understand adaptation to training programmes, assessing fatigue and recovery, and minimising the risk of injury and illness. To this end, technological advancements have enabled the collection of multiple points of data for use in analysis and injury prediction. The full breadth of available data has, however, only recently begun to be explored using suitable statistical methods. Advances in automatic and interactive data analysis with the help of machine learning are now being used to better establish the intricacies of the player load and injury relationship. In this article, we examine this recent research, describing the analyses and algorithms used, reporting the key findings, and comparing model fit. To date, the vast array of variables used in analysis as proxy indicators of player load, alongside differences in approach to key aspects of data treatment—such as response to data imbalance, model fitting, and a lack of multi-season data—limit a systematic evaluation of findings and the drawing of a unified conclusion. If, however, the limitations of current studies can be addressed, machine learning has much to offer the field and could in future provide solutions to the training load and injury paradox through enhanced and systematic analysis of athlete data.

## Key Points

- Football injuries can lead to extended periods of absence from competition, with associated impacts on team performance, as well as financial implications. The relationship between training load and injuries is now a key research and applied focus, but current models and statistical approaches to data analysis fail to sufficiently capture the nuances of this relationship.
- The application of machine learning to the training load and injury relationship is a new but fast growing research area, but there is a lack of consensus regarding which variables to consider for analysis, let alone those subsequently proving to be key in predicting players’ injuries, making it difficult at this time to draw on those studies when choosing which training load variables upon which to focus.
- Although questions remain as to the current utility of machine learning for real-world application, the use of machine learning has great potential to unearth new insights into the workload and injury relationship, if research is expanded to examine multiple seasons’ data, accounts for data imbalance, and uses explainable artificial intelligence.

## Introduction

With technological developments in data collection and storage, football clubs are increasingly *data-driven* [1, 2]. The *multi-camera method* and *electronic performance and tracking systems*,^1^ alongside wearable sensors and use of questionnaires, has allowed practitioners to collect more detailed physical, technical, and psychological data from players [1, 2]. These data can be used to inform scouting, performance analysis, and tactics [1, 3], but increasingly they are being used to better understand the aetiology of injuries [4]. Injuries can lead to extended periods of absence from matches, with associated impacts on team performance, as well as financial implications [1, 4]. As such, the relationship between *training load*^2^ and injuries is now a key focus in football (as it is in all sports). In contrast to other data-centric contexts (e.g. health care; autonomous vehicles), however, comparatively little effort has been invested in understanding football injuries and their prediction using *machine learning*. Indeed, much of the existing injury research has tended to focus on a limited number of training load variables, while the application of multivariate statistical and machine learning methods—despite their obvious utility for understanding complex, multi-dimensional, problems—has been largely ignored [5]. The few studies that have used machine learning techniques to understand and predict football injuries show their potential. The timeliness of using machine learning for sports injury prediction is also highlighted by recent reviews [6, 7]. We complement this work via close examination of research on injury prediction in football, providing details of the approaches employed, along with comparison of methods, data, and results, and by providing recommendations for practitioners. Before closer examination of these studies in football, we first briefly highlight current approaches to understanding the training load and injury relationship and then introduce machine learning and techniques from machine learning with application to understanding the prediction of football injuries. Thus, as well as highlighting the specifics of those studies on football injury, this article should serve to aid readers both from sport science and machine learning communities in their understanding of sports injury articles employing machine learning.

## Training Load and Injuries

Monitoring the load placed on athletes in training (and competition) is a current “hot topic” [8] in sport science, with professional sports teams investing substantial resources to this end [4]. Load monitoring is essential for determining adaptation to training programmes, understanding responses to training, assessing fatigue and recovery, and minimising the risk of injury and illness [8, 9]. Load can be broadly classified into two types: internal and external. Internal training load includes physiological (e.g. heart rate, blood lactate, oxygen consumption) and psychological (e.g. RPE—ratings of perceived exertion, stress, well-being) markers, collected via wearable sensors and questionnaires; external training load includes variables collected via electronic performance and tracking systems (EPTS)—e.g. velocity, acceleration, deceleration, average speed, top speed—as well as numerous other variables, such as power output and weight lifted [4, 8].

Although accumulated evidence that higher training workloads may be associated with greater injury risk has led to the recommendation that workloads should be reduced to minimise injury risk [10–13], the “Workload-Injury Paradox” [10, 11] describes the phenomenon whereby intense workloads may also be associated with injury resilience. Indeed, for sport scientists working full-time in the field, any instruction to reduce workloads for currently healthy players will frequently prove to be unpopular. In seeking to better understand and unpick the key features and components of training load and associated injury risk, several methods have been developed. Banister and colleagues [14] described differences between a positive *fitness* function and a negative *fatigue* function. The “10% rule” [15] describes protection from injury to the extent that week-to-week workload changes do not exceed 10%. The Acute Chronic Workload Ratio (denoted ACWR), developed by Hulin and colleagues [16], is the most popular and well-researched model of the injury process (despite known limitations [17]), describing the ratio of acute (i.e. rolling average of training load completed in the past week) to chronic (i.e. rolling average of training load completed in the past 4–6 weeks) workload. ACWR values exceeding 1.5 have been shown to lead to a 2–4 times greater injury risk over the following week, with an optimal range for ACWR suggested as between 0.85 and 1.35. Session load [18] is the product of RPE of training sessions and the duration of those sessions. “Overtraining syndrome” occurs when session loads exceed a player’s ability to fully recover [18], and the related concept of monotony (i.e. the ratio of the mean and standard deviation of training loads—the sum of all session loads—recorded each week) has been noted as a strong risk factor for injury in studies of skating, basketball, and football [18–20]. Finally, the ratio of internal (e.g. physiological and psychological factors) and external (e.g. data collected via GPS) workload variables [21, 22] has been demonstrated to be important as a predictor of injury.

## Machine Learning

Machine learning is the scientific study of mathematics and statistical models to enable computers to use data to automatically learn and make better decisions from experience [23]. Machine learning has been applied to many areas of science, health care, and finance industries, such as for image detection, cancer detection, stock market prediction, and customer churn prediction [5, 23]. In some areas, such as sport, the effective use of machine learning is in its infancy [5, 24].

The *algorithms* (the “rules” to be followed in calculations) used in machine learning are termed *supervised* learning methods (e.g. regression and classification) and *unsupervised* learning methods (e.g. clustering) [23]. Supervised learning methods are based on *labelled* input and output data (i.e. every piece of input data has a corresponding output—in the case of injury prediction, training load variables would be considered input data; and injury occurrence as output data); unsupervised learning methods are based only on unlabelled input data (i.e. the input data do not have corresponding outputs) [23, 24]. The focus in this paper is on supervised algorithms, especially classification—predicting classes or categories as opposed to continuous values—because injury prediction is commonly based on clearly labelled training data and player injuries. In its simplest form, the task of any machine learning model is to correctly predict injuries (a positive class) and non-injuries (a negative class). Common supervised machine learning algorithms are linear and logistic regression, decision tree, random forest, k-nearest neighbours (often denoted KNN), support vector machine (often denoted SVM), artificial neural networks (often denoted ANN or NN), and “ensemble methods” (e.g. bagging; and boosting) [24]. Of these machine learning algorithms, some are termed *white-box* algorithms (e.g. linear regression, logistic regression, k-nearest neighbours, decision tree); some are termed *black-box* algorithms (e.g. ensemble methods, random forest, artificial neural networks, support vector machine) [25]. White-box algorithms are known as *interpretable* approaches, which are useful, because they present a clear mapping from inputs to outputs, clarifying how analysis decisions are made—and potentially aiding practitioners and clinicians in deriving applied implications from such research. With black-box algorithms, however, this mapping from inputs to outputs is opaque. Thus, with the latter algorithms, additional *post hoc* methods are needed to interpret and understand their results [25, 26]. The key point to note from the above is that all these terms are simply various algorithms that may be used, each of which may perform better or worse under different conditions.

Many real-life machine learning tasks, including injury prediction, are based on *imbalanced* datasets. Imbalanced datasets include a far higher number of negative examples (i.e. non-injuries) than positive examples (i.e. injuries). A problem for machine learning models can then arise, because they tend to learn from those data points present in the highest numbers (in this case, the non-injuries) and subsequently predict those non-injuries well, but fail to predict injuries [27, 28]. To improve the performance of models with such imbalanced data, studies can employ balancing techniques such as oversampling (e.g. to artificially create more injury data points) or undersampling (e.g. to remove non-injury data points), resulting in datasets with a more even balance of non-injuries and injuries. Although each approach has its drawbacks, such a process should lead to machine learning models which favour prediction of neither injury nor non-injury [27, 28].

Classification machine learning models are typically evaluated via a number of fit metrics, some of which, such as accuracy and area under the curve (AUC), are expressed as a single value, while others, such as precision, recall, and specificity, can have different values depending on the choice of the positive class [23, 24]. Assuming that injuries are considered as the positive class and non-injuries as the negative class, accuracy is the ratio of correctly predicted injuries and non-injuries to the total number of observed injuries and non-injuries; precision is the ratio of the correctly predicted injuries to the total number of correctly and incorrectly predicted injuries; recall is the ratio of correctly predicted injuries to the total observed injuries (often described as the true positive rate or sensitivity); specificity is the ratio of correctly predicted non-injuries to the total of observed non-injuries (often described as the true negative rate); and F1-score is the “harmonic” mean (compared to a simple average, this helps to protect against any extreme values) of precision and recall. (As such, this metric is sometimes considered an optimal blend of precision and recall.) These metrics are often expressed in percentages. *AUC* is the probability curve of the true positive rate and false positive rate, with scores close to 1 indicating the best-fitting models [24].

Often the per-class metrics (precision, recall, specificity, and F1-score) are calculated for each class (e.g. injuries and non-injuries) separately and averaged to provide a single overall score. Although this can be reasonable in some instances, the overall score can also be misleading with imbalanced datasets, such as is often the case with football injury data. This is because this overall score tends not to reflect how well the model performs on what is termed the “minority class” (in this case, the injury data, because there tend to be far fewer injury than non-injury data points)—our principal focus of interest. Thus, in the latter case, recall and F1-score *of just the injury class* would be considered particularly useful metrics, while at the same time precision and specificity of both the injury and non-injury data help to protect against drawing conclusions which may then be biased towards the prediction of injuries. Finally, although AUC is often regarded as a very useful evaluation metric, it has also been noted to be misleading with imbalanced data [29]. Studies (including those highlighted in the present article) do not use these metrics in a uniform manner—that is, studies employ some but not all of, and not the same, metrics—as such, comparing studies is far from a simple process.

Extending the above, a typical machine learning study would proceed as follows: data collection, data pre-processing, application of machine learning algorithms (i.e. model training), and model evaluation [30, 31]. Following data collection, data pre-processing can include data cleaning (e.g. missing values imputation, handling outliers, anomaly detection), data transformation (including data normalisation), feature selection (where only a subset of the original data are used in the model), and feature extraction (where new features are created from the original raw data, to perform better within the machine learning algorithm) [31, 32]. This pre-processing stage generally enhances the performance of the machine learning algorithms more than if they were fed with the original raw data [31, 32]. Following data pre-processing, there are two main approaches to evaluate the performance of machine learning models. In the first approach, the dataset is divided into two parts—training data (c. 70–80% of the dataset) and validation data (c. 20–30% of the dataset). This process is termed *train–validation split* (although it is also frequently termed train-*test* split)*.* The training data are fed into a machine learning algorithm (e.g. decision tree, support vector machine, or artificial neural network), resulting in a *trained* model. The predictive performance of this trained model is then subsequently assessed with the validation data. In the second approach, a machine learning model is trained on different subsets of the data and then assessed with further (validation) subsets of the same data. This process is termed *cross-validation*. Regardless of approach, some researchers also set aside a final portion of the dataset as “test” data—here, after validation, the models are applied to the test data to provide a final unbiased estimate of the models’ performance [30–32]. How well the trained model performs with the (validation or) test data is then assessed by means of the evaluation metrics noted above (i.e. accuracy, precision, recall, specificity, F1-score, and AUC) [30]. The purpose of these validation and test processes is to try to reduce *overfitting*—a phenomenon whereby a model is biased towards the data it has been trained on, but has poor predictive performance when applied to previously unseen validation/test data. Machine learning is usually an iterative and cyclical process, such that, depending on the model’s performance, analysts return to earlier stages of the process, to change feature selection, to modify the settings (often called hyperparameters) of their machine learning algorithm (a process termed hyperparameter optimisation), or to try an alternative machine learning algorithm. This entire iterative and cyclic process occurs during the training and validation phases [30–32]. A key point to note from the above discussion is that pre-processing techniques are applied to the training, validation, and test data, but balancing techniques are only applied to the training data. Indeed, balancing of the validation or test data would be undesirable, because assessment of the trained model would not reflect its application and performance with real-world (and unbalanced) data. In following all the preceding steps, the prediction performance of the machine learning model is often assessed and compared against what is termed a *baseline model.* Baseline models may be simple machine learning algorithms or *dummy classifiers* which use simple heuristics such as predicting the most frequent class (i.e. in our case non-injuries). With regard to feature selection, baselines normally include the most basic set of features. These base classifiers vary across studies and are set by the researchers (i.e. there are no fixed baseline criteria that must be adhered to). Ordinarily, researchers also attempt to compare their results with those from similar previous studies, a challenging process with football injury prediction, given the infancy of the area, and (as we note below), the differences in load variables used and evaluation methods employed across these studies. Ultimately, the goal is to derive a model with the best evaluation metrics with the test data. For non-experts, understanding this process is useful when trying to glean the key message from research using machine learning.

## The Application of Machine Learning for Injury Prediction in Football

In Sect. 4.1, we highlight research applying machine learning techniques to football injury prediction, describing the type of injury, the machine learning algorithms employed, the machine learning methodology, and, if mentioned, the important injury predictors (it is worthy of note that not all studies are explicit with regard to the key predictors in their models); Sect. 4.2 (and Tables 1–3) provides a summary.

**Table 1:** Descriptive data for the highlighted papers

	No. of players	No. of injuries	Age group (years)	Injury type	Dataset time span
Rossi et al. [33]	26	21	20–30	Every non-contact	23 weeks
Naglah et al. [35]	21	36	Unreported	Every non-contact	16 months
López-Valenciano et al. [36]	132	32	Unreported	Lower leg muscle	Pre-season + 1 Season
Ayala et al. [37]	96	18	Unreported	Hamstring strain	Pre-season + 1 season
Rommers et al. [39]	734	368	10–15	Acute and overuse	Pre-season + 1 season
Oliver et al. [41]	400	99	10–18	Non-contact lower leg	Pre-season + 1 season
Vallance et al. [42]	40	142	23.6–35.2	Every non-contact	Pre-season + 1 season
Venturelli et al. [43]	84	27	14–18	Thigh muscle strain	Pre-season + 1 season
Kampakis [44]	Unreported	Unreported	Unreported	Not specified	Unreported

Only Oliver et al. [41] and Vallance et al. [42] specifically reported using “male” players. The other papers noted the following: young football players, elite football players, youth players, and/or professional football players

**Table 2:** Training load features in the highlighted papers

*External load*	[33]	[35]	[36, 37] ^*^	[39]	[41]	[42]	[43]	[44]
Exposure							X	
Jumps		X					X	
Distance	X	X				X		X
Accelerations and decelerations	X	X				X		X
DSL (Total weighted impacts above 2 g)	X							
Duration		X				X		
Player load		X				X		X
Speed and velocity		X				X		X
Meterage per minute		X						
Total efforts		X						
High inertial movement analysis		X						
Average metabolic power								X
Dynamic stress load								X
Impacts								X
Energy expenditure								X
Step Balance								X
Dribbling				X				
Sprint				X				
Jumping, moving and balancing				X				
*Internal load —physical data*								
Body mass index	X		X	X	X	X	X	
Fat percentage				X			X	
Step yo-yo test				X			X	
Heart rate		X						
Ratings of perceived exertion (RPE)						X		
*Internal load—psychological data*								
Sleep quality			X			X		
Physical exhaustion			X					
Reduced sense of exhaustion			X					
Sport devaluation			X					
Fatigue, shape, pain, pleasure, worry, satisfaction						X		
*Personal information*								
Height and weight			X	X	X	X	X	
Age	X		X	X	X	X	X	X
Role of the player (Position)/field position	X		X			X	X	X
Previous injury	X		X			X	X	
Minutes played in previous games	X							
Number of games played								
before each training session	X							
Dominant leg	X		X					
Current level of play			X					
Injury details								X
Season stage								X
Activity								X
Phase of play								X
Footwear								X
Surface condition								X
Sitting height, curl-ups, leg length				X				
75% Hop, SLCMJ, SLHD, Y-balance, TJ Knee					X			
ACWR and MSWR of training loads	X							
Neuromuscular training loads				X	X			
Total training load features	55	65	151, 229	29	20	27	18	18

Neuromuscular training loads is an over-arching “feature” which includes multiple variables not explicitly mentioned here

*These two papers included 151 and 229 training load variables, under eight over-arching topics (with the most important ones noted here)

**Table 3:** Model fit for the best-fitting models from each paper

	Machine learning algorithms	Pre-processing techniques	Accuracy (%)	Precision (%)	AUC	Recall (%)	F1-score (%)	Specificity (%)
[33]	Decision tree	*Feature selection*, *oversampling*–SMOTE	–	50	0.76	80	64	–
[35]	SVM	Data normalisation	83.50	–	–	–	–	–
[36]	SmoteBoost	*Oversampling*—SMOTE	–	–	0.75	65.90	–	79.10
[37]	SmoteBoost	*Oversampling*—SMOTE	–	–	0.84	77.80	–	83.80
[39] (a)	XGBoost	Unmentioned	–	85	–	85	85	–
[39] (b)			–	78	–	78	78	–
[41]	Decision tree	Various balancing techniques	–	–	0.66	55.60	–	74.20
[42] (a)*	Random forest	*Missing values imputation*	95.5	92.2	0.92	94.5	–	–
[42] (b)*	XGBoost		97	97	0.97	97	–	–
[43]	Cox regression	Unmentioned	–	–	–	–	–	–
[44] (a)	Supervised principal components analysis	Unmentioned	88.80	55	–	33	–	–
[44] (b)			97.07	19	–	20	–	–

Each paper used a different overall set of model fit metrics. In papers [39, 42] and [44], two key differential approaches (denoted *a* and *b*) were used

^*^This article did not explicitly mention evaluation metrics—we approximated these values from the article’s presented boxplots

### Existing Research

Rossi and colleagues [33] examined non-contact injuries. The authors collected 954 data recordings (each data record held information about players’ daily training load) from 80 training sessions, using 18 training load variables. To account for data imbalance, they employed the “ADASYN” [34] oversampling technique. The authors used *decision trees* as the machine learning algorithm, employed both train–test split and cross-validation approaches, and constructed four baseline models with different combinations of training loads and machine learning models (i.e. logistic regression and random forest). The classification models examined in this study included ACWR, the ratio of mean and standard deviation (MSWR), and the exponentially moving average (EWMA) of each external training load variable (i.e. training load variables collected via GPS) individually, as well as with all training load variables simultaneously. The results demonstrated that a model including all load variables produced the best evaluation metrics when compared with standalone models for ACWR, MSWR, and EWMA. In this model including all load variables, EWMA of previous injuries, EWMA of high-speed running distance, and MSWR of total distance monotony appeared to be the key predictors.

Naglah and colleagues [35] examined non-contact football injuries caused by what they termed high-intensity workouts. (More detailed information is not presented.) The authors initially implemented the k-means classification (an unsupervised classification algorithm) and k-nearest neighbours algorithm on each of 65 training load variables individually using a cross-validation approach, albeit no baseline models are explicitly noted. Subsequently, using those training load variables which were significant in the initial approach simultaneously, and with support vector machine, they reported an *accuracy score of* 83.5%; for comparison, k-means classification with each load variable individually generated accuracy of between 40 and 75%. Overall, a model including all 65 training load variables appeared the most optimal, but further specifics on which individual variables might be most important are lacking.

López-Valenciano and colleagues [36] and Ayala and colleagues [37] examined lower limb muscle injuries [36] and hamstring strains [37], comparing a range of machine learning models using 151 and 229 training load variables, respectively. To account for data imbalance, both studies employed several balancing techniques: random oversampling, random undersampling, and synthetic minority oversampling (SMOTE) [38]. Bagging and boosting machine learning algorithms were tested, in order to select the best performing machine learning model for injury prediction, with both studies using a cross-validation approach and the ADTree machine learning algorithm as a baseline model. The *SmoteBoost* (i.e. a combination of SMOTE and boosting) technique provided the best machine learning model (with 52 [36] and 66 [37] of the load variables). Of the 52 variables found to be important for predicting injury in López-Valenciano and colleagues’ study, three key ones were history of lower extremity muscle injury in the last season, peak torque knee flexor concentric 300 degree dominant leg, and sport devaluation (an aspect of burnout). Of the 66 variables found to be important for predicting injury in Ayala and colleagues’ study, history of hamstring strain injury last season, sleep quality, reduced sense of accomplishment, and range of motion-passive hip flexion with the knee extended-dominant leg appeared to be key variables.

Rommers and colleagues [39] examined both the prediction of (a) total injuries and (b) acute versus overuse injuries. The authors used the XGBoost algorithm to build their machine learning models, employed both train–test (on the whole dataset) and cross-validation (on the training data only) approaches, alongside grid search (a type of hyperparameter optimisation process) as the hyperparameter optimisation process. The authors did not, however, mention any baseline model. This study is notable for being *interpretable* (see following section on black-box models), because Shapley Additive exPlanations (SHAP) [40] was used for interpretation and visualisation. SHAP demonstrated that, of the 29 training load variables examined, the five most important for predicting injuries were age at peak high velocity, body height, leg length, percent body fat, and standing broad jump. For classifying injuries as either acute or overuse, the five most important variables were age at peak high velocity, moving sideways, sitting height, 20-m sprint, and *T* test left (a specific form of sprint test, involving movements forwards and sideways).

Oliver and colleagues [41] examined non-contact lower limb injuries based on “neuromuscular” training loads (using 20 variables). The authors examined the relationship of continuous and categorical training load variables with injuries individually and then used those variables significantly associated with injuries as inputs for multivariate logistic regression. In the latter analysis, only single leg counter movement jump (SLCMJ) peak vertical ground reaction force asymmetry remained a significant contributor to injury. The authors also implemented different ensemble (e.g. bagging, boosting) machine learning algorithms. To account for the data imbalance inherent in this dataset, the authors employed four unspecified balancing techniques. The authors used a cross-validation approach, with the J48 machine learning algorithm as a baseline model. A total of 57 machine learning models were generated, with the bagging machine learning algorithm leading to the best performing model. Across all models SLCMJ asymmetry figured prominently, attesting to its importance. Single leg hop for distance asymmetry, hop and stick (75% hop) asymmetry, knee valgus on the left leg, age, body mass, height, and leg length were also (albeit less so) prominent.

Vallance and colleagues [42] examined non-contact injuries, with data from 245 training sessions, using 27 training load variables. The authors ran analyses with a focus on (a) the upcoming week and (b) the following month, using machine learning with five different sets of training load variables (termed “feature sets”)—each set contained a combination of personal information, plus GPS, physical, and psychological data. The authors used a cross-validation approach, alongside Bayesian optimisation (a type of hyperparameter optimisation process) as the hyperparameter optimisation process, with a baseline model which predicted only non-injuries. Across all analyses, k-nearest neighbours, random forest, decision tree, and XGBoost achieved the best results. The inclusion of personal, GPS, and psychological data to a baseline model (which considered past injuries only) resulted in the most accurate models. For the upcoming week, the best results were achieved using decision tree and random forest, with the following psychological features being the key predictors: RPE, pleasure, and satisfaction. For the subsequent month, the best results were achieved using XGBoost, with the following features being key predictors: RPE, pleasure, satisfaction, pain, physical shape, worry, fatigue, and maximum velocity. The presence of data imbalance in this study was likely somewhat alleviated by the increased number of positive cases (i.e. injuries) occurring with the focus on the upcoming week/month.

Finally, Venturelli and colleagues [43] examined thigh muscle strains in young players using a survival probability model (i.e. evaluation of the time—from the first training load assessment date—players “survived” without injury until occurrence of a first injury) with univariate and multivariate Cox regression on 26 variables. In their multivariate analysis, previous injuries, height, and percentage difference between two kinds of jumps (countermovement jump and squat jump) were found to be significant injury predictors. Further, in an unpublished PhD thesis [44], using various machine learning models with 69 training load variables, supervised principal components analysis outperformed all other machine learning models for injury prediction, but model fits were quite poor.

## Summary of the Research

In sum, Rossi et al. [33], López-Valenciano et al. [36], Ayala et al. [37], Oliver et al. [41] and Vallance et al. [42] implemented various white-box, tree-based machine learning algorithms in their models. Naglah et al. [35], Vallance et al. [42], Venturelli et al. [43], and Kampakis [44] applied black-box machine learning algorithms (support vector machine, artificial neural networks, Cox regression). Rommers et al. [39] also used a black-box model, but to counter the problem of interpretability, employed SHAP to interpret and visualise their results. The majority of articles used techniques such as SMOTE, random undersampling, and random oversampling to counter data imbalance. Further, all articles used cross-validation, although note that Rossi et al. [33] used a prequential evaluation approach (common in *stream* data classification—also noted below), whereby their model was repeatedly tested on incoming (in their case, weekly) small data batches, which were then added to the training data—this approach of evaluation and updating with new data may more closely mirror the real-world experience of practitioners using all available data to predict injuries in real time. Table 1 gives basic descriptive information about each study, including players' ages, types of injury, and time frame—each of these factors could be important in determining which features are selected during machine learning as the most prominent injury predictors.

Table 2 lists the training load variables considered as input features in the studies. Despite some consistency, there is also wide variability in features, meaning that drawing conclusions across studies is complex. Thus, the lack of consensus regarding which features to consider for analysis, let alone those subsequently proving to be key in predicting players’ injuries, makes it difficult at this time for practitioners to rely on these studies when choosing which training load features upon which to focus.

The above notwithstanding, the evaluation metrics in Table 3 appear to demonstrate that overall, the best models for injury prediction are those reported by Rossi et al. [33], Ayala et al. [37], Rommers et al. [39], and Vallance et al. [41]. The work of Rommers et al. [39] and Vallance et al. [42] considered a far greater number of injuries than the other studies, potentially improving prediction. Ayala et al. [37], Rommers et al. [39], and Vallance et al. [42] used boosting-based algorithms, which thus appear to work well in this context. Both Rossi et al. [33] and Ayala et al. [37] used data oversampling, while Rommers et al. [39] and Vallance et al. [42] did not use any data balancing techniques, presumably because of their larger datasets and greater number of positive cases (i.e. injuries).

Overall, although the research highlighted in this article demonstrates the potential of machine learning for bringing new insight to our understanding of injury prediction in football, as readers might observe, there is considerable variability in study design and analysis. More generally, a major concern (and a future research issue) is that the studies examined here are based on data collected across a single season. An important future direction would be to test and refine the developed models on subsequent seasons’ data, with their inherent changes in players, coaches, training, and matches. Indeed, in addition to the above, might a consideration of aspects such as the workload–injury paradox, ACWR, and overtraining syndrome aid in the design of research and analysis plans to make the most of the predictive ability of machine learning models? The paper from Rossi and colleagues [33] is the only one to take the latter approach, drawing on ACWR, MSWR, and EWMA in their machine learning analysis.

Building from the above, although the machine learning techniques employed in the research highlighted above are quite sound, greater detail regarding the machine learning approaches employed would help any objective assessment of their contribution towards better understanding the workload–injury relationship. For example, greater clarity with regard to whether the reported evaluation metrics are “per-class” or “averaged” would be important—only Rossi and colleagues [33] explicitly mentioned recall and precision of their models for injury and non-injury data separately. Further, as injury datasets likely have large amounts of missing and unclean data, greater detail regarding the various pre-processing techniques employed (e.g. in relation to any missing values, different data imputation techniques required, balancing, and clarity regarding all types of demographic data, and internal and external load variables) would be important for drawing conclusions and guiding future work. Here, only López-Valenciano et al. [36] and Ayala et al. [37] gave a complete account of all the various pre-processing techniques they used in their research.

With the above said, researchers would be well advised to consider several key points before employing machine learning models. The first is to clearly define the task—often drawing on the needs and preferences of football practitioners. For example, is the interest simply in raw predictions, probabilities, or in examining specific features impacting injuries? Second, with regard to data compilation and pre-processing, practitioners at football clubs are likely to have varied sources of data, often in unique formats, such that great care should be taken to avoid errors when compiling such data into one final dataset. Third, we would recommend ensuring that input data are examined in relation to injuries occurring after collection of those input data—i.e. such that the model may predict injuries in the future (e.g. in one day’s time or in seven days’ time). That is, any training and input data from the same day that an injury has occurred should be disregarded, because such data may be confounded by the injury occurrence. Fourth, with regard to data pre-processing, given the longitudinal nature of football injury datasets, it would make more sense to replace missing values on a player-by-player basis, rather than across the whole dataset, as well as using interpolation for this purpose for some features. Similarly, data balancing might also be conducted on a player-by-player and/or season-by-season basis. Finally, as noted above, changes of coaches, managers, players, and training regimes across seasons mean that the underlying distribution and quality of data will vary from season to season. Traditional machine learning algorithms assume that the underlying distribution of the data is the same. To counter this problem, a focus on what is termed stream learning may help to better understand and interpret machine learning models with multi-season data. What the preceding lines suggest is that future machine learning research in this area could be well served by drawing from current expertise, insight, and understanding from sport science and sport practitioners.

## Conclusion

Machine learning for football injury prediction is a new but fast growing research area. Machine learning approaches can help expand the focus from univariate models, to create a better understanding of the relative influence of various (physical and psychological) aspects of training load on injury risk. In this article, part of our aim was to highlight (and to an extent de-mystify) the machine learning process. Machine learning is simply an analytical technique, but its power lies in its ability to work so eloquently with such a vast array of load variables. Although this can offer greater flexibility over analysis with more simplified models (e.g. using ACWR), the myriad ways machine learning can be employed can also lead to difficulty in synthesising the current research evidence into an overall, unified, conclusion. Indeed, there remain questions as to the utility of these models for real-world application. The use of *white-box* machine learning algorithms in a number of the present articles should aid understanding and application. *Black-box* models may, however, offer better predictive performance, despite being difficult to interpret and understand. The latter issue of interpretability can be addressed using explainable artificial intelligence approaches, like SHAP [40], Local Interpretable Model-agnostic Explanations [45], and partial dependency plots [46, 47]. Despite its infancy, coupled with the limitations we have noted, machine learning for understanding the workload–injury relationship in football is clearly a method whose time has come. By expanding the focus to multiple seasons’ data, accounting for data imbalance, and using explainable artificial intelligence, machine learning should help to unlock new insights into the workload–injury relationship.

## Data Availability

Not applicable.

## Footnotes

1 Electronic performance and tracking systems include optical-based camera systems, local positioning systems (LPS), and global positioning systems (GPS).

2 Training load is variously described as workload, load variables, training load variables, features, input features, or risk factors.
